# Photoacoustic monitoring of oxygenation changes induced by therapeutic ultrasound in murine hepatocellular carcinoma

**DOI:** 10.1038/s41598-021-83439-y

**Published:** 2021-02-18

**Authors:** Mrigendra B. Karmacharya, Laith R. Sultan, Chandra M. Sehgal

**Affiliations:** Department of Radiology, Perelman School of Medicine, University of Pennsylvania, 3620 Hamilton Walk, Philadelphia, PA 19104 USA

**Keywords:** Cancer, Tumour angiogenesis

## Abstract

Hepatocellular carcinoma (HCC) is a highly vascular solid tumor. We have previously shown that ultrasound (US) therapy significantly reduces tumor vascularity. This study monitors US-induced changes in tumor oxygenation on murine HCC by photoacoustic imaging (PAI). Oxygen saturation and total hemoglobin were assessed by PAI before and after US treatments performed at different intensities of continuous wave (CW) bursts and pulsed wave (PW) bursts US. PAI revealed significant reduction both in HCC oxygen saturation and in total hemoglobin, proportional to the US intensity. Both CW bursts US (1.6 W/cm^2^) and the PW bursts US (0.8 W/cm^2^) significantly reduced HCC oxygen saturation and total hemoglobin which continued to diminish with time following the US treatment. The effects of US therapy were confirmed by power Doppler and histological examination of the hemorrhage in tumors. By each measure, the changes observed in US-treated HCC were more prevalent than those in sham-treated tumors and were statistically significant. In conclusion, the results show that US is an effective vascular-targeting therapy for HCC. The changes in oxygenation induced by the US treatment can be noninvasively monitored longitudinally by PAI without the use of exogenous image-enhancing agents. The combined use of PAI and the therapeutic US has potential for image-guided vascular therapy for HCC.

## Introduction

Hepatocellular carcinoma (HCC) is the most common primary liver malignancy and is a leading cause of cancer-related death worldwide^1^. The highest HCC incidence rates have been reported in Southeast Asia and sub-Saharan Africa^2^, with a 5-year survival rate as low as 18%^3^. These solid tumors are very vascular and their development critically depends on the formation of new blood vessels^4^. They are also highly active metabolically and utilize accelerated aerobic glycolysis to produce ATP for tumor growth by reprograming cell metabolism for enhancing glucose uptake^5^. A highly elevated expression of glycolytic enzymes has been shown to be associated with HCC tumorigenesis^6^. This glycolytic paradigm shift, involving the accumulation of lactate in the cellular environment, promotes angiogenesis and an accompanying increase in blood flow to the HCC. Accordingly, there is significantly augmented arterial hepatic blood flow to the carcinoma when compared to the normal liver parenchyma^7^.

The high dependence of HCC on angiogenesis for tumor survival and growth has provided a strong rationale for vasculature targeting therapies^8^, including transarterial chemoembolization (TACE)^9^. TACE is the standard of care for patients with large or multinodular HCC, preserved liver function with no cancer-related symptoms, and no evidence of vascular invasion or metastases^10^; there may, however, be a heterogeneous therapeutic response^11^. Also endothelial growth factor (VEGF) inhibitors have been proposed for antivascular HCC treatment^12^. However, unexpected and potentially life-threatening systemic toxicities including severe thrombotic and hemorrhagic complications have been reported^13^.

Recently, the use of ultrasound (US) as an antivascular agent to disrupt tumor vasculature has been demonstrated in various cancer models^14–18^. The ultrasonic waves upon propagation through tumors exert significant heating and mechanical effects that disrupt the tumor vasculature^19^. Bioeffects of US treatment on tumors can be enhanced by the concurrent intravenous administration of microbubbles, nanoparticles, and other exogenous materials^20–23^. While vascular measurements are valuable they do not provide information on tissue oxygenation, which is an important parameter that determine HCC treatment outcome. The significance of tumor oxygenation in monitoring therapeutic efficacy, or in assessing tumor vascularity, has been recognized in various cancer models, including HCC^24–27^. Baseline tumor oxygen saturation is often regarded as an important functional parameter for tumor therapy^28^.

Photoacoustic imaging (PAI) is a contrast-agent-free biomedical imaging modality that maps tumor oxygenation in real time^29^. PAI detects endogenous light-absorbing chromophores such as hemoglobin and generates a three-dimensional (3D) high-resolution parametric map of tissue oxygen saturation^30^. Tumor oxygenation has been demonstrated to be an early marker of tumor response for predicting treatment efficacy^31,32^. In this study we evaluated the potential of PAI as an imaging modality to monitor antivascular US therapy in a murine model of HCC. With the increased oxygenated blood flow in the neovasculature of metabolically active HCC tumors, utilizing low-intensity US as an antivascular agent, we hypothesized that PAI could quantitatively assess the efficacy of US therapy by measuring the decreases in tumor oxygenation over time. We used PAI to assess changes in tumor oxygenation following antivascular US therapy with a range of ultrasonic intensities in continuous wave (CW) bursts and pulsed wave (PW) bursts modes. Three different US treatment groups with spatial-average-temporal-average intensities (I_SATA_) of 0.32, 0.8, and 1.6 W/cm^2^, along with various peak pressure amplitudes, were investigated and compared. The effects of US therapy on thermal dose in tumors were also examined.

## Results

### PAI showed decrease in oxygen saturation after US treatment

**Figure 1:**
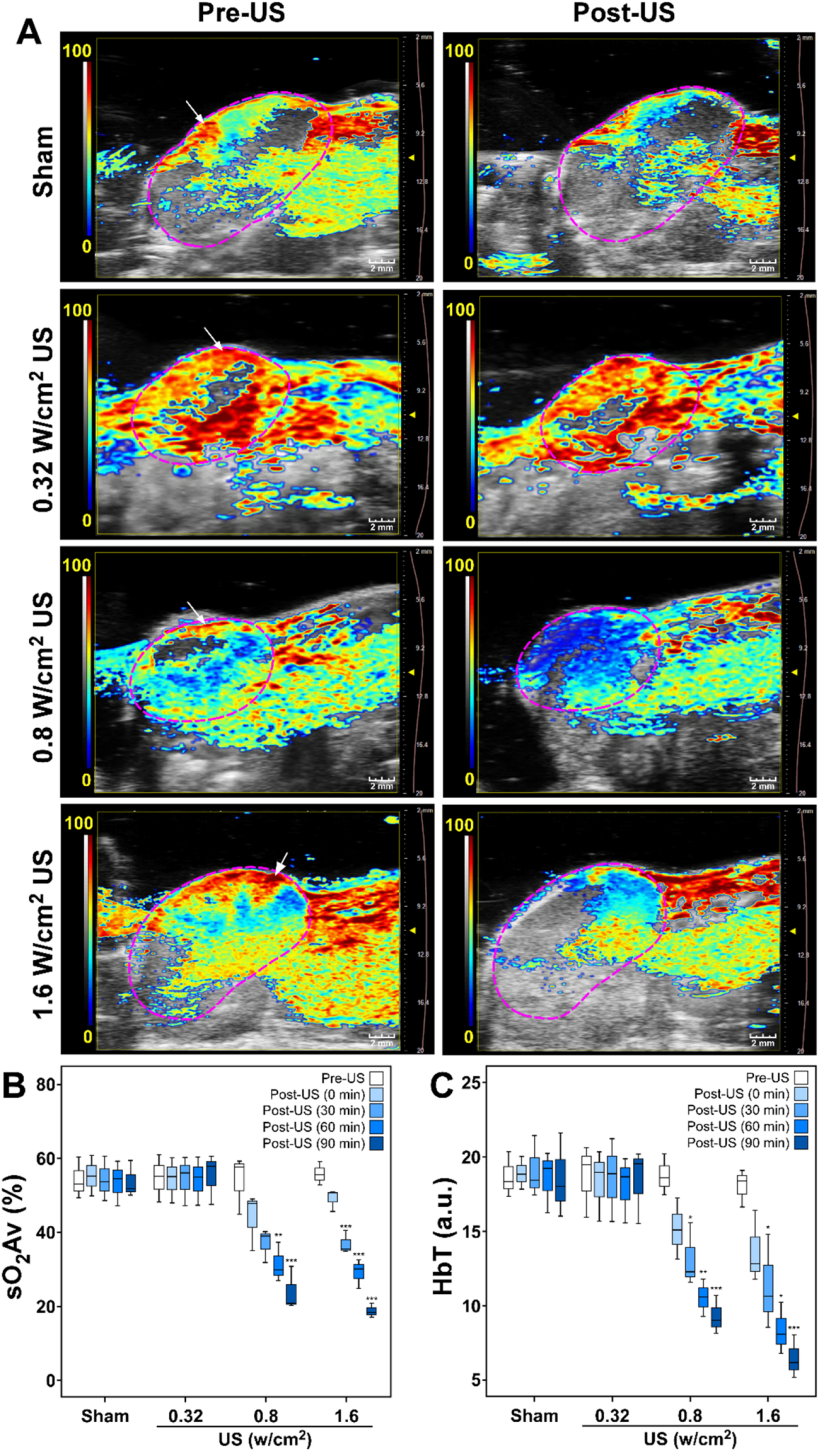
Photoacoustic assessment of HCC tumor oxygenation before and after US treatment. Three different US treatment groups with I_SATA_ of 0.32 W/cm^2^ (peak pressure = 0.23 MPa), 0.8 W/cm^2^ (peak pressure = 0.36 MPa), and 1.6 W/cm^2^ (peak pressure = 0.23 MPa) were investigated and compared. (**A**) Representative two-dimensional PA images of tumors displaying blood oxygen saturation maps with co-registered grayscale B-mode images acquired before (Pre-US) and 90 min after (Post-US) ultrasound therapy. Oxygenation level ranges from 0% (dark blue) to the 100% (dark red). White arrows show high PA signal in the periphery of the tumor. The boxplots display the minimum, first quartile, median, third quartile, and the maximum of the oxygenation measurements for (**B**) sO_2_ average (sO_2_Av) and (**C**) total hemoglobin (HbT) in the whole tumor volume. Mean values of sO_2_Av or HbT in the whole tumor volume for the sham, 0.32, 0.8, and 1.6 W/cm^2^ US treatment groups were calculated and compared, Pre-US versus Post-US. *, **, and *** represent statistical significance of *p* ≤ 0.05, *p* ≤ 0.01, and *p* ≤ 0.001, respectively. Scale bar 2 mm.

Figure 1A shows blood oxygen saturation on a scale of blue to red colors within the tumor area enclosed within the dotted line (Fig. 1A). Before US treatment all HCC tumors exhibited high blood oxygen saturation level, represented by yellow to red colors, especially at the periphery of the tumor (indicated by arrows in Fig. 1A). Treatment with 0.8 or 1.6 W/cm^2^ US substantially reduced the blood oxygen saturation level after treatment, in the range represented by black to blue colors. Treatment with 0.32 W/cm^2^ US, on the other hand, did not show any noticeable change in blood oxygen saturation following the treatment. The sham-treated group also did not show any change in oxygenation.

Quantitatively, sO_2_Av and HbT decreased markedly with US treatment. The decrease was dependent on both intensity and time (Fig. 1B,C). Treatment with 0.8 W/cm^2^ US decreased sO_2_Av from 57.69 to 47.81, 39.00, 29.87, and 20.91% after 0, 30, 60, and 90 min of treatment, respectively. Similarly, it decreased HbT from 18.60 (arbitrary unit) to 15.08, 12.27, 10.58, and 9.02 (arbitrary unit) after 0, 30, 60, and 90 min after treatment respectively. Decrease in sO_2_Av and HbT by 0.8 W/cm^2^ US at 60 and 90 min post-treatment were all statistically significant. A similar decrease in sO_2_Av and HbT was observed at 1.6 W/cm^2^. Treatment with 1.6 W/cm^2^ US decreased sO_2_Av (%) from 55.59 to 50.70, 35.48, 30.15, and 18.35%; and HbT (arbitrary unit) from 18.38 to 12.81, 10.64, 8.06, and 6.19 (arbitrary unit) after 0, 30, 60, and 90 min after treatment respectively. Decrease in sO_2_Av and HbT by 1.6 W/cm^2^ US at 30, 60 and 90 min post-treatment were all statistically significant. As opposed to the US-treatment groups at 0.8 and 1.6 W/cm^2^ intensity, sO_2_Av and HbT did not change over time and remained at levels comparable to those before treatment in 0.32 W/cm^2^ US and sham-treated control groups.

### Power Doppler (PD) images showed decrease in vascularity after US treatment

**Figure 2:**
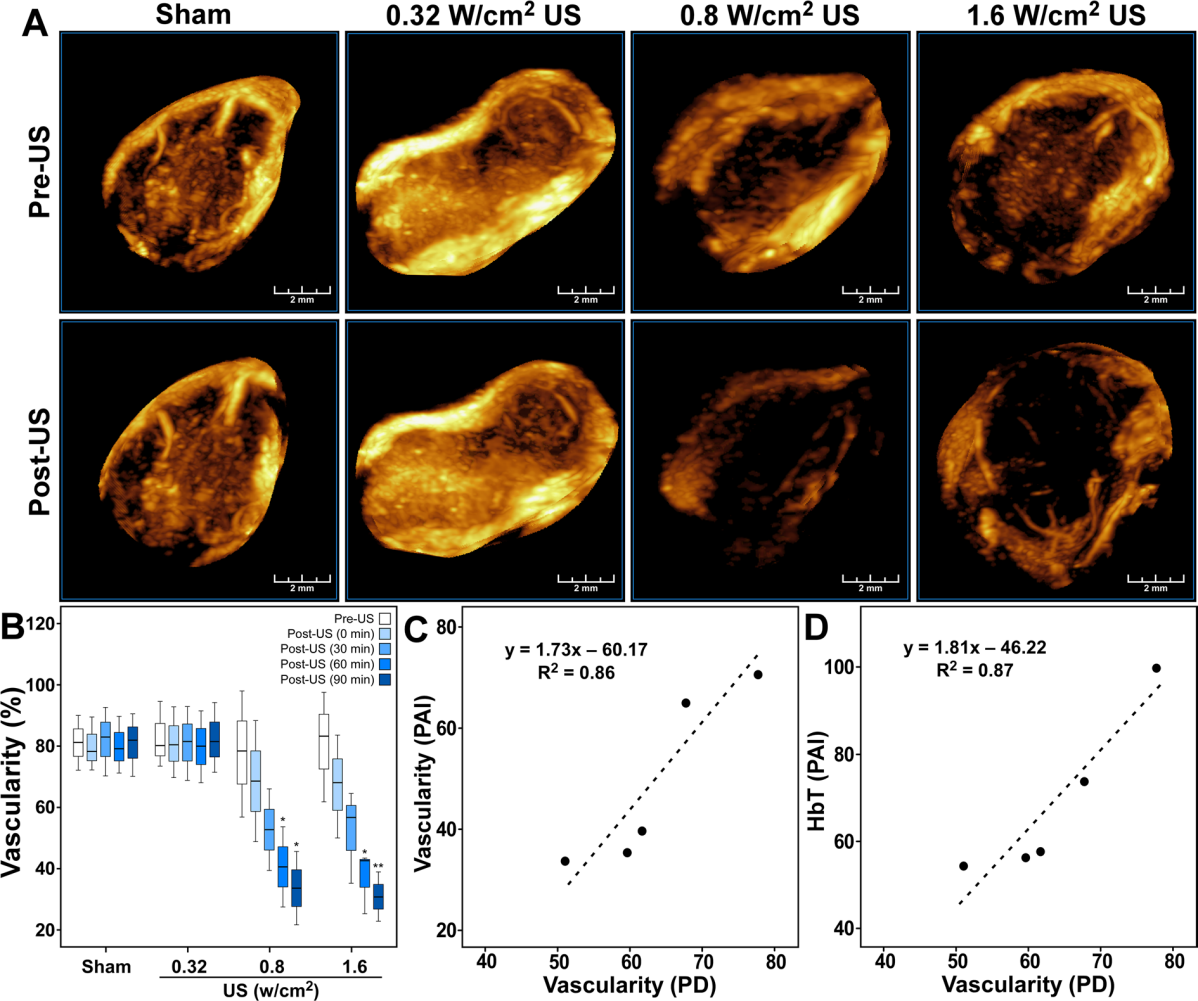
Power Doppler (PD) assessment of HCC tumor vascularity before and after US treatment. (**A**) Three-dimensional maximum-intensity-projection (MIP) images of the tumors displaying PD signal before (Pre-US) and 90 min after (Post-US) ultrasound therapy from representative cases. Scale bar 2 mm. (**B**) Boxplots showing the minimum, first quartile, median, third quartile, and the maximum of percentage vascularity in whole tumor volume. Mean values of percentage vascularity in the whole tumor volume for the sham, 0.32, 0.8, and 1.6 W/cm^2^ US treatment groups were calculated and compared, Pre-US versus Post-US. Statistical significance is indicated by *, **, and *** for *p* ≤ 0.05, *p* ≤ 0.01, and *p* ≤ 0.001 respectively. (**C**) Graph showing the correlation between the change in the ratio of colored pixels to the total number of pixels in the ROI obtained from PAI with vascularity obtained from power Doppler. (**D**) Graph showing the correlation between change in total hemoglobin (HbT) with vascularity obtained from power Doppler.

Sonographic assessment of HCC tumors by PD imaging showed well-developed vasculature inside tumors in the maximum-intensity-projection (MIP) images (Fig. 2A): a dense network of tumor vasculature was observed inside tumors before US treatment. PD signal was substantially reduced following treatment with therapeutic US at the intensities of 0.8 or 1.6 W/cm^2^. The changes were most noticeable in the central region of the tumors. Treatment with 0.32 W/cm^2^ US did not show a change in PD signal, and the images remained comparable to those obtained before the treatment. The sham treatment group did not show any change.

Quantitatively, tumor vascularity decreased significantly after US treatment (Fig. 2B). When measured after 0, 30, 60, and 90 min of US treatment, the percentage tumor vascularity decreased from 78.41% to 68.57, 52.73, 40.58, and 33.64% at 0.8 W/cm^2^; and from 80.87% to 67.22, 52.16, 37.11, 28.51% at 1.6 W/cm^2^ acoustic intensity. The decreases in vascularity at 60 and 90 min after therapy for both 0.8 and 1.6 W/cm^2^ intensities were statistically significant. Sham-treated control groups and 0.32 W/cm^2^ US groups did not show any decrease in tumor vascularity after treatment.

A comparison of the ratio of colored pixels to the total number of pixels in the ROI calculated from PAI (which is a measure the vascularity within the region) with the vascularity in the tumor volume measured from power Doppler showed a high correlation, with Pearson correlation coefficient (R) of 0.93 and *p* < 0.025 (Fig. 2C). Similarly, there was a strong correlation between the total hemoglobin concentration (HbT) of the tumor calculated from PAI with vascularity measured from power Doppler (R = 0.93 and *p* < 0.025, Fig. 2D).

### Histological analysis confirmed increase in hemorrhage after US treatment

**Figure 3:**
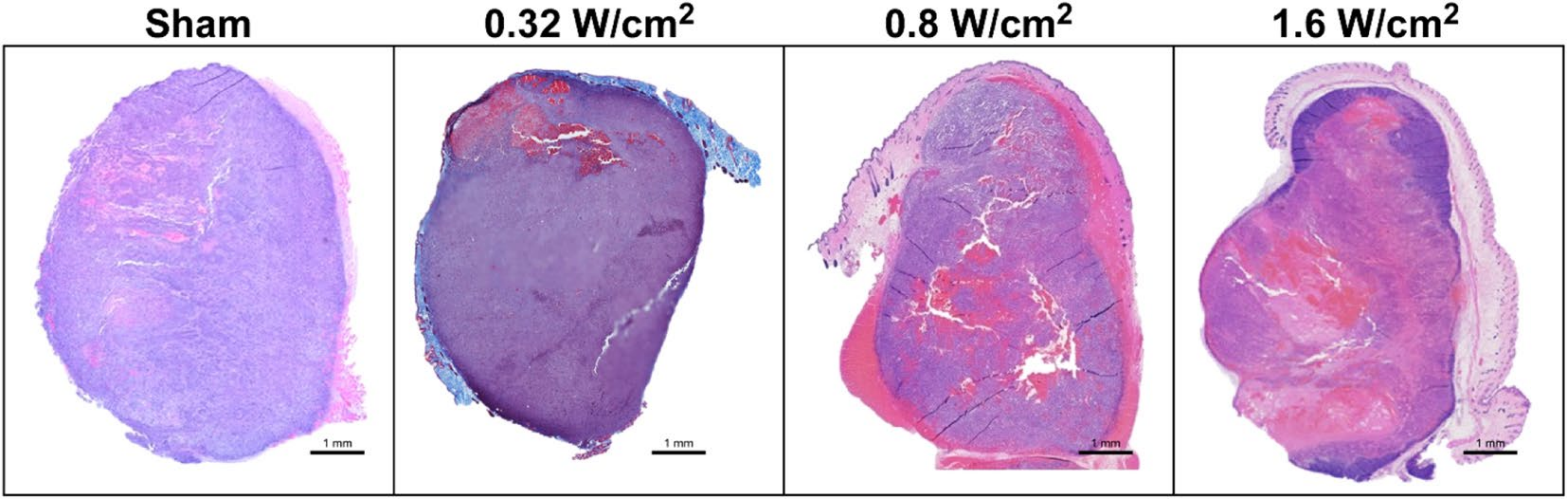
Histologic evaluation of murine HCC tumors treated with US. H&E-stained images taken from sham-treated and US-treated (I_SATA_ = 0.32, 0.8, or 1.6 W/cm^2^) tumors collected after treatment. Pink color represents hemorrhagic area. Scale bar 1 mm.

Histological analysis of the H&E-stained tumor slices showed that US-treated tumors were more hemorrhagic than the sham-treated tumors (Fig. 3). While only 6.6 ± 1.1% of the total tumor area was hemorrhagic in the sham-treated controls, the hemorrhagic area in 0.8 W/cm^2^ and 1.6 W/cm^2^ US-treated groups increased to as high as 40.8 ± 3.9% and 49.1 ± 2.7% respectively. The difference in the hemorrhagic area between the sham control and the US-treated groups was statistically significant (*p* < 0.01). In the 0.32 W/cm^2^ group, merely 9.1 ± 1.3% of the tumor area was hemorrhagic and not statistically different from the sham controls (*p* > 0.05).

### US treatment induced tumor heating

**Figure 4:**
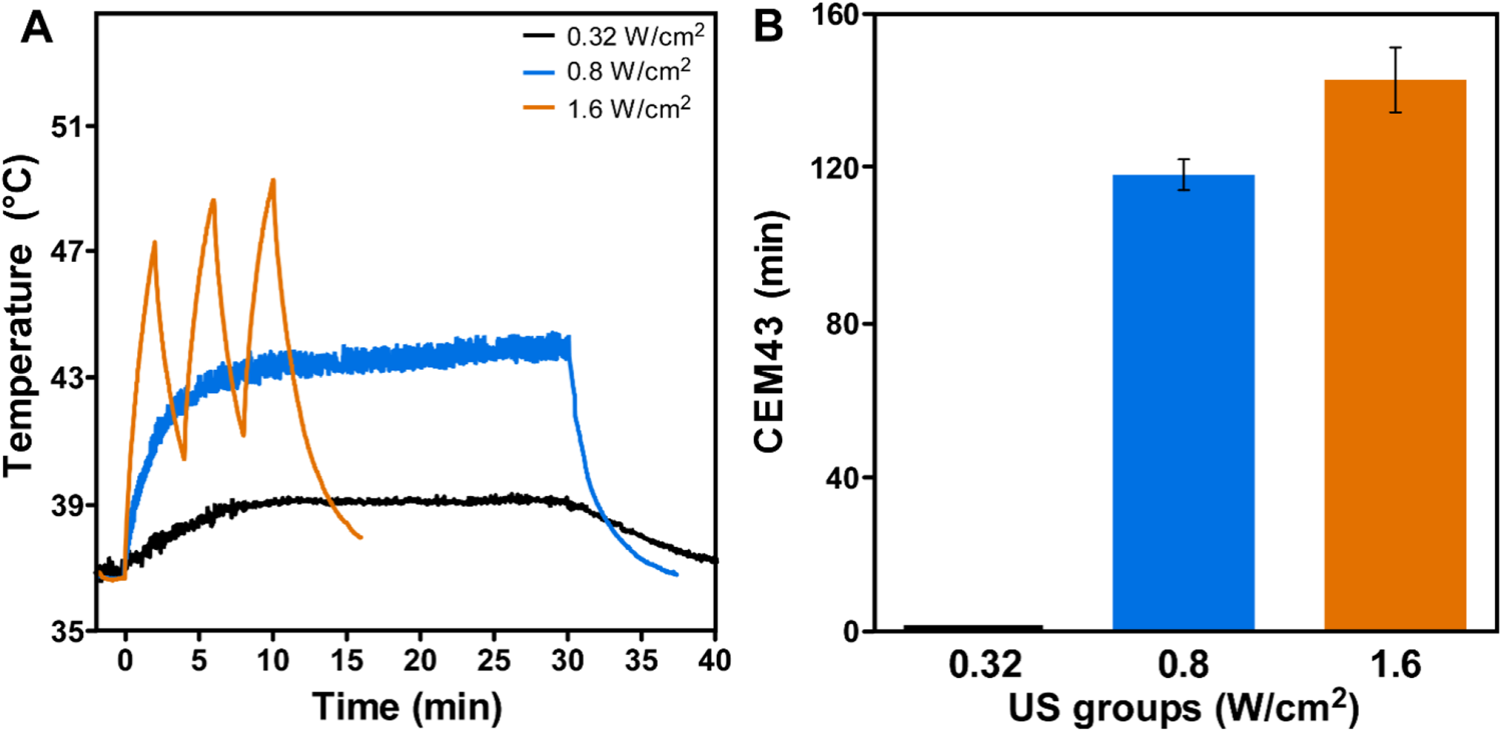
Tumor temperature and thermal dose (CEM43) measurement. (**A**) Graphs showing tumor temperature in live mice during US treatment at 0.32, 0.8 and 1.6 W/cm^2^. Black line represents 0.32 W/cm^2^ US; blue line represents 0.8 W/cm^2^ US; and orange represents 1.6 W/cm^2^ US. (**B**) Graph showing thermal dose (CEM43) measured for three different ultrasound treatment groups.

The tumor temperature measured in live mice showed that treatment with 0.8 and 1.6 W/cm^2^ US, but not with 0.32 W/cm^2^ US, delivered significant thermal dose (CEM43) to the insonated tumor (Fig. 4A). Thermal doses (CEM43) delivered by therapeutic US at 0.8 and 1.6 W/cm^2^ US were 117.2 ± 4.1 min and 142.1 ± 8.4 min, respectively, compared to 0.20 ± 0.01 min at 0.32 W/cm^2^ US (Fig. 4B).

### US-induced bioeffects were correlated with sonication intensity I_SATA_

**Figure 5:**
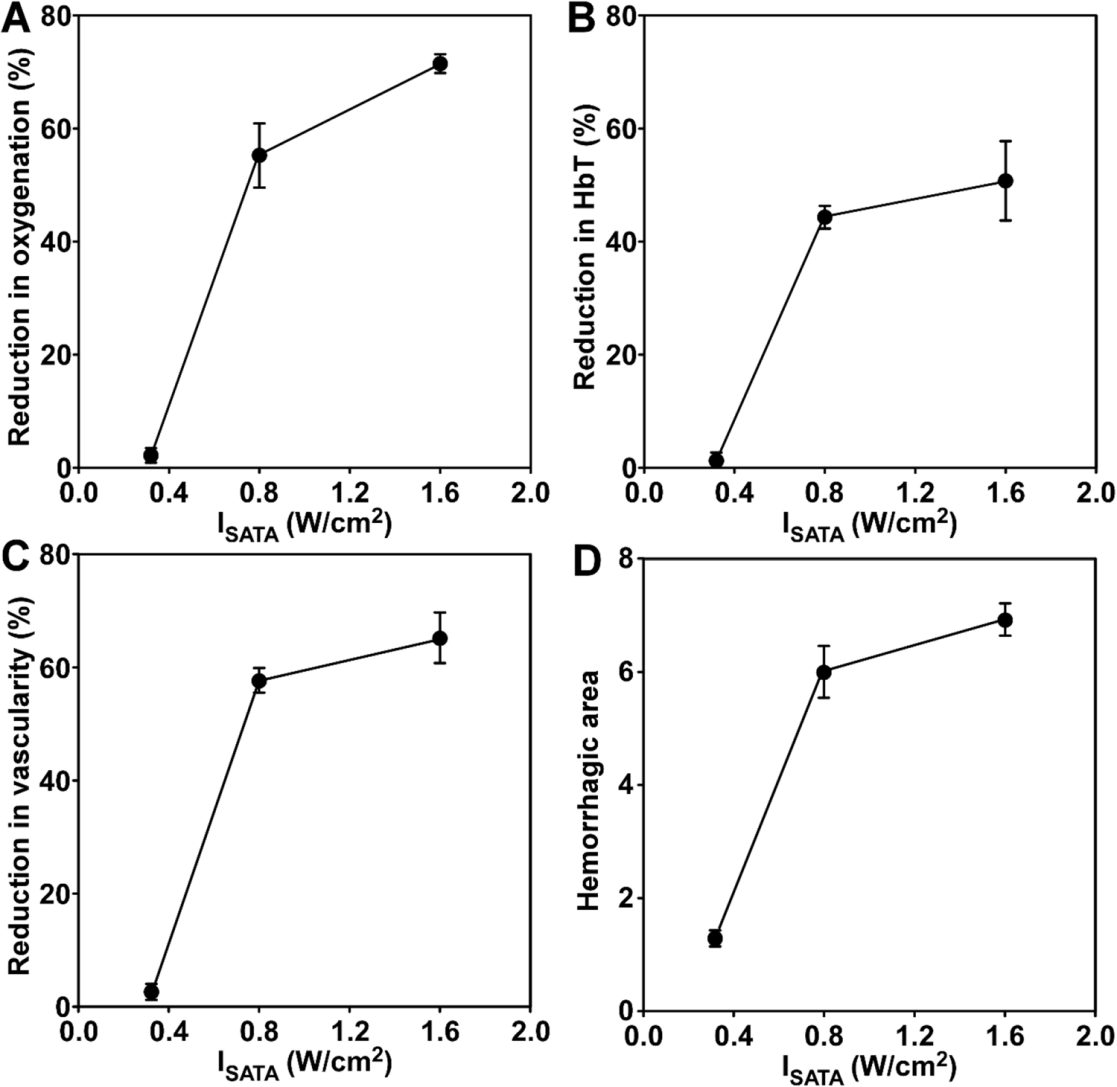
Effects of US intensity on tumor oxygenation, vascularity, and hemorrhage. Percentage reduction in sO_2_Av (**A**), HbT (**B**), PD vascularity (**C**) with respect to the pre-treatment condition, and hemorrhage area relative to the sham-treated controls (**D**) were plotted as a function of sonication intensity (I_SATA_).

The percentage reduction in PAI measurements, namely, sO_2_Av and HbT, increased with US intensity (I_SATA_), with a tendency to level off at higher intensity (Fig. 5A,B). Vascularity changes measured by PD and increases in hemorrhage area relative to sham followed the same trend as PA measurements (Fig. 5C,D). US treatment at 0.32 W/cm^2^ intensity did not produce any observable changes in sO_2_Av, HbT, vascularity, or hemorrhage, while both 0.8 and 1.6 W/cm^2^ intensities produced significant changes in these measurements compared to pre-treatment values (*p* < 0.001). Consistently, the reduction in sO_2_Av, HbT, and vascularity, and the hemorrhage area relative to the sham controls all showed a near-linear correlation with CEM43 (Supplementary Figure S1).

## Discussion

PAI has received increasing attention as a state-of-the-art imaging technique in cancer biology^33^ as it offers opportunities to understand tumor pathophysiology and the biological mechanisms underlying cancer therapy. In PAI, the photoacoustic signal from oxygenated and deoxygenated hemoglobin generates a high-resolution tomographic map of oxygen saturation in real time^34^.

In the current study, PAI was used to assess the changes in tumor oxygenation and vascularity induced by US therapy in murine HCC. Several trends related to HCC oxygenation and vascularity changes induced by US were observed. The results show that blood oxygen saturation within HCC tumors is non-uniformly distributed. Most parts of the tumor were well oxygenated while in smaller regions of the tumor there were lower blood oxygen saturation levels. This non-uniformity in blood oxygen saturation distribution within the tumor volume is likely due to spatial heterogeneity in the tumor microenvironment caused by variations in tumor physiology and vascularity. When a tumor grows its internal neovasculature and blood supply develops non-homogeneously such that some parts of the tumor are poorly vascularized compared to others which are relatively well vascularized^35^. Because tumor neovasculature is aberrant, immature, disorganized, and has high structural heterogeneity, some parts of the tumor are metabolically more active than others. This results in the non-uniform distribution of blood oxygen saturation within the tumor volume. Earlier studies have reported a considerable heterogeneity in tumor blood flow, which is often vigorous in some regions of the tumor and slow in others^36^.

When treated with US, blood oxygen saturation markedly decreased in tumors. After US treatment with either 0.8 or 1.6 W/cm^2^, blood oxygen saturation in tumors was reduced significantly, with most parts of the tumors showing oxygenation at the lower end of the scale. Insonation not only decreased the average blood oxygen saturation but also reduced the total hemoglobin in tumors in a time-dependent manner. Reduction in tumor blood oxygen saturation is due to dysfunctional intra-tumor arterial supply resulting from the disruption of blood vessels by US. It has been shown that direct disruption of the tumor vasculature leads to a rapid inhibition of tumor blood flow^37^, which elicits several biochemical events which further amplify and exacerbate the antivascular effects in tumors. Consistent with the PAI results, obliteration of tumor blood vessels after US treatment was observed with PD imaging. PD signals were markedly reduced after US treatment, and in the histological studies formation of large hemorrhagic pools was observed. Importantly, unlike in some previous studies, microbubbles were not injected intravenously prior to insonation, yet the antivascular bioeffect was still observed.

The current study shows that the bioeffects of US treatment were not transitory; rather, the antivascular effects persisted, and increased progressively for at least 90 min post-insonation. Tumor insonation at 0.8 and 1.6 W/cm^2^ led to statistically significant decreases in the PAI parameters and PD vascularity, demonstrating their potential for monitoring the physiological and vascular changes in HCC tumors. The continued post-insonation response showed that tumors remained partially functionally active, suggesting that US therapy at these low intensities was not completely ablative but selective in damaging the tumor vasculature. To our knowledge, it has not been previously demonstrated that therapeutic ultrasound can induce oxygenation changes in HCC tumors. The dose of ultrasound required to induce these changes is also yet to be reported. Therefore, the current study focused on demonstrating the ability of PAI to assess tumor oxygenation changes induced by US treatment. It should be emphasized here that investigating tumor growth kinetics extending days or weeks after the US treatment would be important, but such studies would be most appropriate only after the dose–effect relationships are better understood, and the ability of PAI to monitor oxygenation changes has been confirmed. The motivation for this research was to lay the foundation for measuring biological outcomes, including tumor growth. HCC tumor neovasculature is known to be excessively leaky^38^ and the fragilities in the structural and functional integrity of these vessels provide a vulnerable target for disruption by low-intensity US. Interestingly, the vascular disruption is progressive as it occurred gradually over time, however, the underlying biological and physiological processes responsible for this change are yet to be determined.

In this study it was observed that therapeutic US at 0.8 W/cm^2^ in PW bursts mode and 1.6 W/cm^2^ in CW bursts mode, but not at 0.32 W/cm^2^ in PW bursts mode, caused a significant decrease in oxygen saturation and vascularity of the tumors. The different treatment protocols provide insights into the underlying biophysical mechanisms that could be playing a role. Both 0.32 W/cm^2^ in PW bursts mode and 1.6 W/cm^2^ in CW bursts mode used the same amplitude US pressure of 0.23 MPa, but they produced markedly different effects on HCC. While CW bursts-mode treatment resulted in a marked reduction in HCC oxygenation, PW bursts mode did not. For a fixed US frequency, peak pressure amplitude is proportional to the mechanical index of US^39^ which determines the likelihood of cavitation effects^19^. The observation that two modes of therapeutic treatment with same pressure amplitude produced different effects is indicative that cavitation is not a significant contributor to the bioeffects observed in this study.

The temporal-average intensity I_SATA_, on the other hand, expresses the ultrasonic intensity delivered to any tissue averaged over the exposure time and is most closely associated with thermal index^40^. The magnitude of thermal bioeffects depends on the total acoustic output power and is directly associated with I_SATA_. Our data on tumor temperature measurements by US demonstrated that 1.6 W/cm^2^ CW bursts US, with lower peak pressure amplitude than that of 0.8 W/cm^2^ PW bursts US, delivered greater CEM43 and also had greater therapeutic effects on HCC. At I_SATA_ of 1.6 W/cm^2^, CEM43 was 142.1 ± 8.4 min, versus 117.2 ± 4.1 min at I_SATA_ of 0.8 W/cm^2^. Similarly, sonication with 0.32 W/cm^2^ US, with peak pressure amplitude comparable with that of 1.6 W/cm^2^ US, could deliver only a minimal CEM43 of 0.2 ± 0.01 min, and did not produce any observable therapeutic effects. The therapeutic effects of US, whether measured by PAI, PD imaging, or histology, when displayed as a function of sonication intensity (I_SATA_) followed a similar pattern to that of CEM43. In this context, the results suggest that the observed antivascular US effects are most likely dominated by thermal effects; non-thermal cavitation effects of US cannot, however, be ruled out. Further, it should be noted that CEM43 for ablative therapies is on the order of 240 to 540 min^41^. Although the equivalent cumulative durations in minutes in this study are shorter than those reported for ablative therapies, the thermal dose is sufficient to induce disruption of the fragile and leaky neovasculature of HCC observed in this study. This is consistent with previous research that showed that CEM43 of 50 min significantly decreased tumor vasculature in human prostate tumor (PC3) xenografts grown in mice^42^.

It should be noted that although the results are encouraging, PAI is in its early stages of development and is subject to depth-dependent optical and ultrasound attenuation effects. The time gain compensation (TGC) of the PA signal accounts for this effect, at least to a first approximation, but penetration depth of PAI continues to be a limitation and remains an active area of research^43^.

In conclusion, noninvasive and real-time assessment of blood oxygen saturation changes by PAI may lead to new US therapies of HCC. Furthermore, effective monitoring of kinetic changes in oxygen saturation and neovasculature post-insonation could be of significance in understanding structural and functional hemodynamic changes following the US treatment. PAI represents a novel approach for noninvasive assessment of tumor oxygenation, eliminating the need for image-enhancing contrast agents, and could play a significant role in monitoring and guiding vascular HCC therapies.

## Materials and methods

### Animals

The animal studies were approved by the Institutional Animal Care and Use Committee. Adult male athymic nude mice weighing 25–35 g were purchased from Charles River Laboratories (Wilmington, MA, USA), and were accommodated in metabolic cages under controlled environmental conditions (25 °C and a 12-h light/dark cycle). Mice had free access to standard powdered and pelleted food and tap water ad libitum. All mice were studied during light cycles.

### Cell culture

Mouse hepatoma cells Hepa1-6 (ATCC CRL-1830) were purchased from American Type Culture Collection (Manassas, VA, USA). The cells were maintained in Dulbecco’s modified Eagle’s medium (DMEM) supplemented with fetal bovine serum (Gibco, Gaithersburg, MD, USA) to a final concentration of 10%, 100 U/mL penicillin and 100 μg/mL streptomycin in a water-saturated atmosphere of 5% CO_2_ at 37 °C. All experiments were carried out 24 h after the cells were seeded.

### Induction of HCC and US treatment

HCC were developed in immuno-deficient athymic nude mice using xenograft models where 1 × 10^6^ Hepa1-6 cells were implanted subcutaneously in the right flank. The growth of HCC tumors was monitored and each tumor was imaged and treated with US when grown approximately 10–15 mm wide. Each mouse was transferred to an acrylic box and general anesthesia was induced with 1–2% isoflurane (Isosol, Halocarbon Laboratories, River Edge, NJ, USA) and 100–200 mL/min oxygen. The animal was then placed on a heated platform (38 °C) in supine position, and anesthesia was maintained via a nosecone. In three treatment groups (n = 3 in each group), the tumors were sonicated for 6 min (on-time) with low-intensity long-tone-burst non-focused sinusoidal plane-wave ultrasound (frequency = 2.8 MHz) generated by a single-element plane-disk transducer (diameter = 15 mm) utilizing either CW bursts or PW bursts modes. For the CW bursts mode, ultrasound were driven in three 2:2 min on:off cycles with I_SATP_ = 1.6 ± 0.002 W/cm^2^, I_SATA_ = 1.6 W/cm^2^, and peak pressure amplitude = 0.23 MPa. The rationale for using the 2 min on and off cycles for CW bursts mode was to prevent heating of the transducer surface. For the PW bursts modes, 1000-ms pulses of ultrasound were driven at 1:4 s on:off cycles (20% duty cycle). Two different intensities were used for PW bursts mode ultrasound, viz.,

i. I_SATP_ = 1.6 ± 0.002 W/cm^2^, I_SATA_ = 0.32 W/cm^2^, peak pressure amplitude = 0.23 MPa; and
ii. I_SATP_ = 4.0 ± 0.003 W/cm^2^, I_SATA_ = 0.8 W/cm^2^, peak pressure amplitude = 0.36 MPa.

The ultrasound parameters (summarized in Table 1) were calculated as described previously^44^. In the sham-treated control group (n = 3), all experimental procedures were identical to the treated groups except that the US therapy probe was not turned on. Each animal was imaged before and after therapy by PAI (Vevo LAZR, FUJIFILM VisualSonics, Toronto, ON, Canada) and PD (VevoLAB, FUJIFILM VisualSonics, Toronto, ON, Canada) to assess changes in oxygenation and vascularity, respectively, induced by the US treatment.

**Table 1:** Summary of the US parameters.

Mode	Frequency (MHz)	Pulse length (ms)	Duty cycle	Intensity, I (W/cm^2^)^b^		Peak pressure, P (MPa)^c^
				I_SATA_	I_SATP_	
	2.8	CW^a^	1.0	1.6	1.6	0.23
	2.8	1000	0.2	0.32	1.6	0.23
	2.8	1000	0.2	0.8	4.0	0.36

^a^Continuous wave bursts with three 2:2 min on:off cycles.

^b^Determined by measuring radiation pressure.

^c^Calculated by using the equation I = P^2^/2ρc, where ρ = density, and c = speed of sound.

### PA image acquisition and analysis

3D-PAI imaging of the entire tumor was performed before and 0, 30, 60, and 90 min after US therapy. The images were acquired using a 13–24-MHz broadband LZ250 transducer with axial and lateral resolutions of 100 and 235 μm, respectively. The VevoLAZR imaging system uses a tunable laser with 4- to 6-ns pulses of peak energy 45 ± 5 mJ and repetition frequency of 20 Hz. The system’s dynamic range for PAI is 70 dB with signal to noise ratio of 20 ± 10 dB. The time gain compensation (TGC) was optimized for each animal according to the size and position of the tumor to compensate for the depth-dependent attenuation of the photoacoustic signal. All other imaging parameters including TGC were kept fixed between pre- and post-treatment. PAI was performed in the OxyHemo mode of the scanner. In this mode, PA signals are acquired at 750 and 850 nm and are unmixed spectrally for deoxygenated and oxygenated hemoglobin by exploiting their differential light absorption spectra^45^. The equation expressing the absorption coefficient of blood as a linear combination of the absorption from the two kinds hemoglobin is solved for the signals at the two wavelengths to determine oxygenated [HbO_2_] and deoxygenated [Hb] hemoglobin concentrations. Oxygen saturation, sO_2,_ is determined by the ratio [HbO_2_]/([Hb] + [HbO_2_]). Unmixing of the PA signals is performed pixel-wise to create a parametric map of sO_2_. The PA images are superimposed in color on grayscale B-mode ultrasound images acquired simultaneously with PAI.

3D PA images consisting of stacks of images in contiguous parallel planes were acquired by moving the transducer with a stepper motor in steps of 0.167 mm with persistence = 8. The 3D images were analyzed for sO_2_. This involved outlining the region of interest (ROI) defining the tumor in each image of the 3D stack. Grayscale B-mode ultrasound images were used to guide the drawing of the tumor margin. For each ROI, sO_2_ average (sO_2_Av) and total hemoglobin (HbT) were measured.

sO_2_Av is the sum of all oxygenated pixels divided by the oxygenated and deoxygenated pixels within the region of interest (ROI). It represents the average blood oxygen saturation within the ROI^46^. HbT, the average hemoglobin concentration calculated from the pixels with both oxygenated and deoxygenated hemoglobin signal, represents the total blood volume within the ROI. The ratio of colored pixels to the total number of pixels in the ROI is a measure the vascularity within the region.

### Power Doppler (PD) imaging and analysis

PD images of tumors were acquired in 3D mode at a step size of 0.167 mm using the same sonographic transducer that was used for PAI. 3D-PD imaging was performed before and 0, 30, 60, and 90 min after US therapy. Imaging presets (gain = 18 dB; high sensitivity; 100% power; 21-MHz transmit frequency; and high line density) and TGCs were optimized for each animal and fixed during the studies. ROIs were drawn around the tumor in each frame of the 3D dataset and analyzed for percentage vascularity over the tumor volume using VevoLAB imaging software (FUJIFILM VisualSonics, Toronto, ON, Canada).

### Histochemical staining and analysis

At the completion of each study, the mouse was euthanized, a necropsy was performed, and the tumor was harvested for histologic examination. Tumors were preserved in 10% phosphate-buffered formalin for 48–72 h before being transferred to 50% ethanol, embedded in paraffin, and finally processed for histological examination with hematoxylin and eosin (H&E) stain. The tumor slices were examined microscopically, images were acquired, and the percentage area of hemorrhage was recorded using ImageJ software^47^.

### Tumor temperature and thermal dose measurement

Tissue heating by therapeutic US was measured for each US treatment protocol using the approach described earlier^17^. Briefly, temperature was measured by a fine-wire thermocouple (0.08-mm diameter; Omega Engineering Inc., Stamford, CT, USA) placed under the exterior surface of the tumor in mice during the therapy. The thermocouple was aligned perpendicular to the US probe and placed about 1 to 2 mm outside the 15-mm-wide US beam. A schematic of the tumor temperature measurement setup is given in Supplementary Figure S2. Temperature was recorded every second for each US treatment protocol. Thermal dose (TD) was computed by the formula^48,49^.

$${\text{TD}} = \mathop \int \limits_{0}^{t} R^{{\left( {T - 43} \right)}} dt,$$

where t = time (min); R = 2 for temperature T ≥ 43 °C; and R = 4 for T < 43 °C. Thermal dose at temperature ≥ 43 °C is usually expressed in terms of cumulative equivalent minutes at 43 °C (CEM43) and is recognized as the most commonly used dosimetry parameter^50^.

### Statistical analysis

The acquired PAI and PD data were quantified as mean ± SEM, and statistical analyses were performed to test the significance of any post-insonation changes using a one-way analysis of variance (ANOVA) followed by Tukey’s *post-hoc* test (IBM SPSS Statistics, IBM Corp., Armonk, NY, USA). Vertical boxplots representing the five-number summary of a set of data (the minimum, first quartile, median, third quartile, and the maximum) were calculated and plotted. Prior to the ANOVA test, the Shapiro–Wilk test was performed to test data normality. A *p*-value > 0.05 was used to accept the data as normally distributed. Pearson correlation coefficients^51^ were performed to determine, post-tumor therapy, whether there were significant associations between changes in PAI recordings of percentage oxygenated blood in the tumor and the PD recordings of tumor vascularity. A similar analysis was used to determine whether there was a correlation between change in the PAI recordings of total hemoglobin (HbT) and the PD measurements of tumor vascularity. Statistical significance was represented as *** for *p* ≤ 0.001, ** for *p* ≤ 0.01 and * for *p* ≤ 0.05, and ns (not significant).

### Ethics statement

The authors confirm that all methods were carried out in accordance with relevant guidelines and regulations. The authors confirm that the study was carried out in compliance with the ARRIVE guidelines (http://www.nc3rs.org.uk/page.asp?id=1357).

## Supplementary information

Supplementary Information The online version contains supplementary material available at 10.1038/s41598-021-83439-y.

Supplementary information.
