# Supplementary material for: Photoacoustic monitoring of oxygenation changes induced by therapeutic ultrasound in murine hepatocellular carcinoma
Source: Sci Rep. 2021 Feb 18;11:4100. doi: 10.1038/s41598-021-83439-y (PMC7893035; doi:10.1038/s41598-021-83439-y)
Supplement: Supplementary file 1 — Supplementary information. [file 41598_2021_83439_MOESM1_ESM.docx]

**Photoacoustic monitoring of oxygenation changes induced by therapeutic ultrasound in murine hepatocellular carcinoma**

**Authors**: Mrigendra B. Karmacharya^§^, Laith R. Sultan^§^, and Chandra M. Sehgal*

§ These authors have contributed equally in this study.

**Authors Affiliation**: Department of Radiology, Perelman School of Medicine, University of Pennsylvania, 3620 Hamilton Walk, Philadelphia, PA 19104.

***Corresponding Author**: Chandra M. Sehgal, Department of Radiology, Perelman School of Medicine, University of Pennsylvania, 3620 Hamilton Walk, Philadelphia, PA 19104, USA. Tel: 215-573-4963; Fax: 215-898-6115; Email: [Chandra.Sehgal@uphs.upenn.edu](mailto:Chandra.Sehgal@uphs.upenn.edu)

**Supplementary Figures**

**
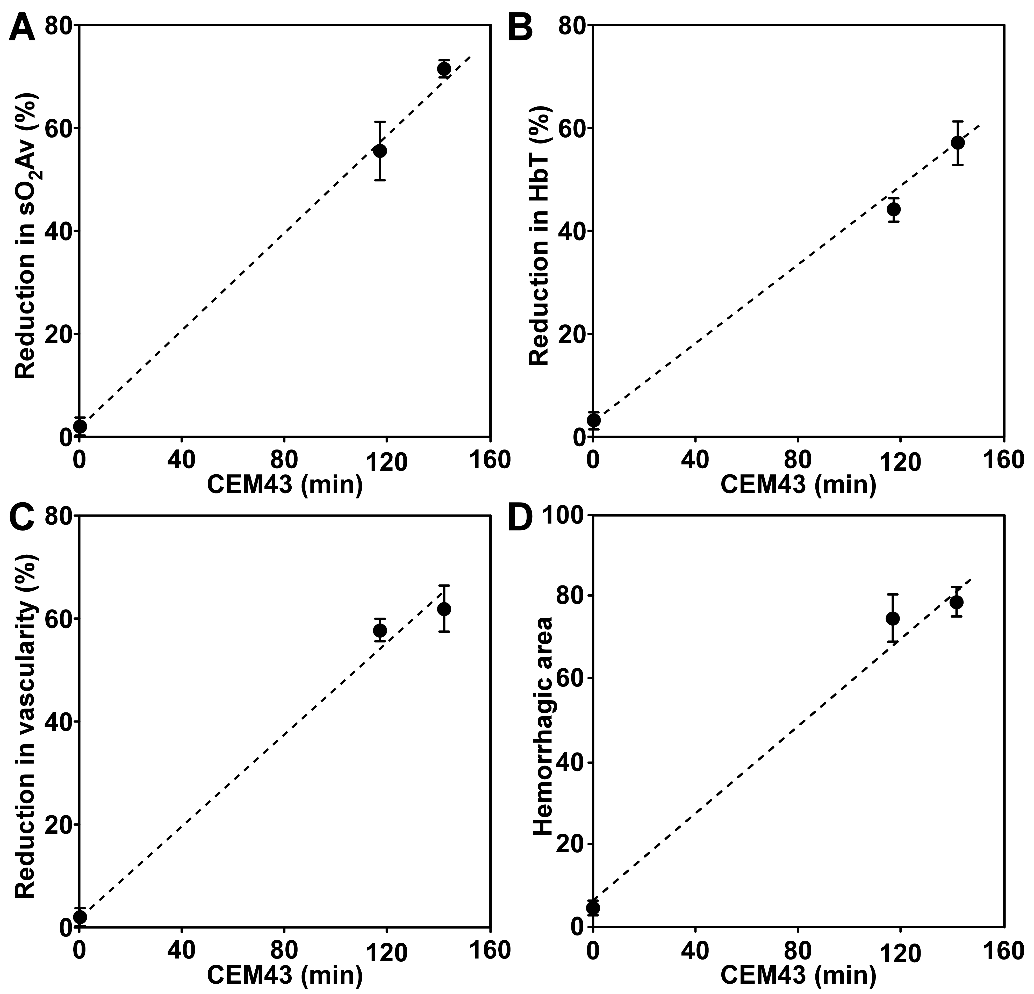
**

**Supplementary Figure S1: Correlation between CEM43 and tumor oxygenation, vascularity, or hemorrhage.** Percentage reduction in sO_2_Av (**A**), HbT (**B**), PD vascularity (**C**) with respect to the pre-treatment condition, and hemorrhage area relative to the sham-treated controls (**D**) were plotted as a function of the thermal dose (cumulative equivalent minutes at 43°C, CEM43).

**
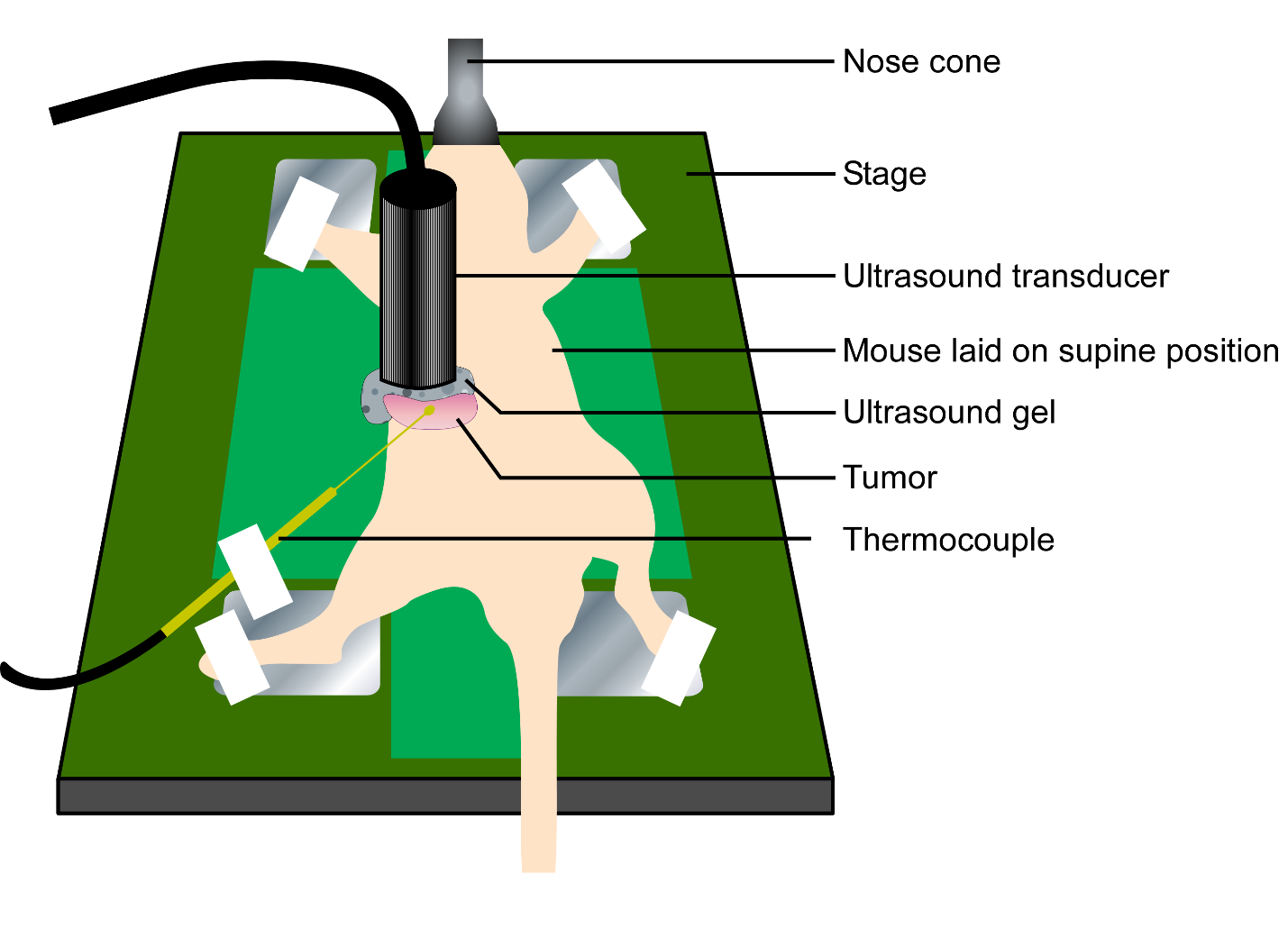
**

**Supplementary Figure S2: Schematic showing the experimental setup.** Note that the thermocouple is placed on the tumor surface perpendicular to the US probe and lies outside the US beam.
